# Exercise and epigenetic ages in older adults with myeloid malignancies

**DOI:** 10.1186/s40001-023-01145-z

**Published:** 2023-05-30

**Authors:** Kah Poh Loh, Chandrika Sanapala, Marielle Jensen-Battaglia, Anish Rana, Michael B. Sohn, Erin Watson, Nikesha Gilmore, Heidi D. Klepin, Jason H. Mendler, Jane Liesveld, Eric Huselton, Marissa LoCastro, Martha Susiarjo, Colleen Netherby-Winslow, AnnaLynn M. Williams, Karen Mustian, Paula Vertino, Michelle C. Janelsins

**Affiliations:** 1 James P. Wilmot Cancer Institute, Rochester, NY USA; 2 Division of Hematology/Oncology, Department of Medicine, University of Rochester Medical Center, 601 Elmwood Avenue, Box 704, Rochester, NY 14642 USA; 3 Department of Public Health Sciences, University of Rochester Medical Center, Rochester, NY USA; 4 School of Medicine and Dentistry, University of Rochester, Rochester, NY USA; 5 Department of Biostatistics and Computational Biology, University of Rochester Medical Center, Rochester, NY USA; 6 Department of Psychology, Princeton University, Princeton, NJ USA; 7 Division of Cancer Control, Department of Surgery, University of Rochester Medical Center, Rochester, NY USA; 8 Wake Forest Baptist Comprehensive Cancer Center, Medical Center Blvd, Winston-Salem, NC USA; 9 Department of Environmental Medicine, University of Rochester Medical Center, Rochester, NY USA; 10 Department of Biomedical Genetics, University of Rochester Medical Center, Rochester, NY USA

**Keywords:** DNA methylation, Epigenetic clock, Mobile health, Exercise intervention, Geriatric hematology, Myeloid malignancies

## Abstract

**Background:**

Older adults with myeloid malignancies are susceptible to treatment-related toxicities. Accelerated DNAm age, or the difference between DNA methylation (DNAm) age and chronological age, may be used as a biomarker of biological age to predict individuals at risk. In addition, cancer treatment can also lead to accelerated DNAm age. Exercise is a promising intervention to reduce or prevent functional, psychological, and cognitive impairments in older patients with myeloid malignancies, yet there is little evidence of the effects of exercise on DNAm age. We explored (1) the associations of accelerated DNAm age with physical, psychological, and cognitive functions at baseline; (2) changes in DNAm age from baseline to post-intervention; and (3) the associations of changes in accelerated DNAm age with changes in functions from baseline to post-intervention.

**Methods:**

We enrolled older patients with myeloid malignancies to a single-arm pilot study testing a mobile health (mHealth) exercise intervention that combines an exercise program (EXCAP^©®^) with a mobile application over 2 cycles of chemotherapy (8–12 weeks). Patients completed measures of physical, psychological, and cognitive functions and provided blood samples for analyses of DNAm age at baseline and post-intervention. Paired t-tests or Wilcoxon signed rank tests assessed changes in DNAm ages, and Spearman’s correlation assessed the relationships between accelerated ages and functions.

**Results:**

We included 20 patients (mean age: 72 years, range 62–80). Accelerated GrimAge, accelerated PhenoAge, and DunedinPACE were stable from baseline to post-intervention. At baseline, DunedinPACE was correlated with worse grip strength (r = -0.41, p = 0.08). From baseline to post-intervention, decreases in accelerated GrimAge (r = -0.50, p = 0.02), accelerated PhenoAge (r = − 0.39, *p* = 0.09), and DunedinPace (r = − 0.43, *p* = 0.06) were correlated with increases in distance walked on 6-min walk test. Decreases in accelerated GrimAge (r = − 0.49, *p* = 0.03), accelerated PhenoAge (r = − 0.40, *p* = 0.08), and DunedinPace (r = − 0.41, *p* = 0.07) were correlated with increases in in grip strength.

**Conclusions:**

Among older adults with myeloid malignancies receiving chemotherapy, GrimAge and PhenoAge on average are stable after a mHealth exercise intervention. Decreases in accelerated GrimAge, accelerated PhenoAge, and DunedinPACE over 8–12 weeks of exercise were correlated with increased physical performance. Future trials assessing the effects of exercise on treatment-related toxicities should evaluate DNAm age.

*Trial registration* Clinicaltrials.gov identifier: NCT04981821.

**Supplementary Information:**

The online version contains supplementary material available at 10.1186/s40001-023-01145-z.

## Background

Myeloid malignancies, such as acute myeloid leukemia (AML) and myelodysplastic syndrome (MDS), most commonly occur in adults aged ≥ 60 years [1–3]. Studies have demonstrated that up to 73% of older patients with myeloid malignancies receiving chemotherapy have physical, psychological, and cognitive impairments prior to and during chemotherapy [4–10]. These impairments can lead to reduced quality of life (QoL), treatment interruptions, and reduced survival [4, 7, 11–14]. Behavioral interventions such as an exercise program to prevent or reduce these impairments can improve outcomes in this vulnerable population.

Aging is a heterogeneous process, and chronological age does not always accurately represent underlying physiologic age [15]. Individuals can age at different rates and experience faster (accelerated) or slower (decelerated) aging, compared to their chronological age [16]. Several methods are available to measure physiologic age, which includes cognitive age, physical fitness, biological age, perceived age, and the frailty index [16]. DNA methylation (DNAm) age is a promising biomarker of biological age [17]. DNAm is a biological process whereby methyl groups are added to CpG sites (cytosine nucleotide followed by a guanine) on DNA molecules [18, 19]. DNAm age is estimated based on weighted average of methylation levels at specific CpG sites [20–24]. Although DNAm age is highly correlated with chronological age [15, 21, 25], it is a more physiologic reflection of biological age, accounting for interactions between the genome, environment and epigenome [15, 26]. The difference between DNAm age and chronological age is suggested to provide a measure of biologic aging. In the general population, both DNAm age (without adjusting for chronological age) and ‘accelerated DNAm age’ (after adjusting for chronological age) are associated with functional decline, cognitive decline, frailty, morbidity, and mortality [20, 27, 28].

Older adults with myeloid malignancies are susceptible to treatment-related toxicities. Accelerated DNAm age may be used as a biomarker to predict individuals at risk. In addition, cancer treatment can also lead to accelerated DNAm age through epigenetic alterations [21, 29–31]. In a previous study, patients with breast cancer who received radiation and chemotherapy experienced greater accelerated DNAm age from pre- to post-treatment (6.2–25.6 years) compared to non-cancer controls (change of less than 1 year over a 2- to 7-year period) [30]. Several algorithms are available to calculate DNAm age (e.g., GrimAge [20], PhenoAge [23], Horvath Age [21, 22], Hannum Age [24], and DunedinPACE) [32]. First generation epigenetic clocks (e.g., Horvath Age, Hannum Age) correlate with chronological age and second generation epigenetic clocks (e.g., GrimAge, PhenoAge) better reflect biological age [33]. Specifically, GrimAge and PhenoAge are strongly associated with functional decline, frailty, morbidity, and mortality in the general population [20, 27]. Because these first and second generation epigenetic clocks measure aging-related change in DNAm accumulated across the life course, they may not be sensitive enough to detect the effects of intervention [34]. DunedinPACE is a rate measure rather than a clock therefore allowing quantification of the changes in the pace of DNAm age in the context of an intervention [34]. A prior study also showed that a lifestyle intervention slowed DunedinPACE [34]. Therefore, we focused on DNAm GrimAge, DNAm PhenoAge, and DunedinPACE [21].

Exercise is a promising intervention to reduce or prevent functional, psychological, and cognitive impairments in older patients with myeloid malignancies [35–38]. In a cross-sectional study, exercise is inversely correlated with accelerated DNAm age [39]. Only one previous study has evaluated DNAm age in a prospective non-randomized, single-arm exercise trial of older adults with hematologic malignancies; it showed that DNAm age decreased in 3 of 10 patients from baseline to post-intervention [40]. We previously demonstrated that a mobile health (mHealth) exercise intervention is feasible, usable, and safe in older adults with myeloid malignancies receiving outpatient chemotherapy over two cycles in a single-arm pilot study [41]. Patients maintained their physical, psychological, and cognitive functions from baseline to post-intervention. In the current study, we study the following aims: (1) the associations of accelerated DNAm age (focusing on Grim Age, PhenoAge, and DunedinPACE) with physical, psychological, and cognitive functions at baseline; (2) changes in DNAm age from baseline to post-intervention; and (3) the associations of changes in accelerated DNAm age with changes in functions from baseline to post-intervention.

## Methods

### Study design, setting, and participants

We conducted a single-arm pilot study of older patients with myeloid malignancies recruited from an academic cancer center [University of Rochester Medical Center/Wilmot Cancer Institute, Rochester, New York, USA)]. Details of the study have been previously reported [41]. Briefly, we included patients aged ≥ 60 years with a myeloid malignancy receiving outpatient-based chemotherapy who were able to walk four meters, had a physician-verified Eastern Cooperative Oncology Group (ECOG) Performance Status between 0 and 2, had no medical contraindications to exercise per the treating oncologist, and were able to provide informed consent. We excluded patients with a platelet count of 10,000 per microliter or less in their most recent complete blood count if they did not receive platelet transfusion. The University of Rochester Research Subjects Review Board approved this study. All participants provided informed consent.

### Study intervention

The Geriatric Oncology-Exercise for Cancer Patients (GO-EXCAP) intervention is an integrated mHealth exercise intervention that combines an exercise program [Exercise for Cancer Patients (EXCAP^©®^)] with a mobile application (app). EXCAP^©®^ is an individually tailored, low to moderate intensity, home-based exercise program consisting of progressive walking and resistance band exercises, delivered by an American College of Sports Medicine (ACSM)-certified exercise physiologist [42]. The mobile app has a patient interface for data entry and an online dashboard assessed by study personnel and exercise physiologists to monitor data.

### Study procedures

After obtaining informed consent, patients completed demographics and baseline measures. Clinical data were obtained by study staff from the electronic medical record. Participants also provided non-fasting blood samples. To obtain baseline step count, patients were provided with an activity tracker (Garmin Forerunner^®^ 35) to wear for 4–7 days prior to start of the intervention. Study participants then met with an ACSM-certified exercise physiologist to receive exercise intervention training, as well as instructions from the study team for mobile app use. They were provided with an EXCAP^©®^ exercise kit (three therapeutic bands and exercise instruction manual), Garmin activity tracker, and a tablet with the mobile app.

Participants performed the exercises at home and entered data on exercises (daily steps and resistance band) and symptoms into the mobile app over two cycles of chemotherapy (i.e., 8 to 12 weeks). The exercise physiologists and study team communicated with the participants through the remote portal and/or by phone and provided tailored feedback regarding intervention progress. At post-intervention, similar measures including blood samples were collected. Post-intervention step counts were collected for 4–7 days during the final week of the intervention.

### Measures

Clinical outcomes included physical function (self-reported and objectively assessed), fatigue, depressive symptoms, cognition, and quality of life.

#### Physical function

Physical function was assessed with the Short Physical Performance Battery (SPPB), virtual SPPB (added during the COVID-19 pandemic), 6-minute walk test (6MWT), and handgrip strength. The SPPB is a valid [43] three-component objective assessment used to evaluate physical function in older adults. It ranges from 0 to 12; higher scores indicate better physical function [44]. The virtual SPPB utilizes the same scoring system as the SPPB and assesses patient self-reported ability to perform the SPPP components [45]. The 6MWT is an assessment of aerobic capacity and functional endurance. The test measures distance walked in six minutes [46]. The handgrip dynamometer was used to assess upper extremity muscle strength. Assessments were performed in an alternating bilateral sequence, with three measurements taken per arm.

We also collected Katz Activities of Daily Living (ADL) and Lawton Instrumental ADL (IADL). The Katz ADL measures independence in six self-care activities (e.g., bathing, ambulating) with scores ranging from 0 to 6, with lower scores indicating greater dependency. The Lawton IADL assesses independence in seven self-care activities that are more complex (e.g., preparing meals, managing finances). Each question rated is on a three-point Likert scale with total scores ranging from 0 to 14, with lower scores indicating greater dependence.

#### Fatigue

Fatigue was measured using the Brief Fatigue Inventory (BFI). The BFI consists of nine items with scores ranging from 0 to 11, with higher scores indicating greater fatigue [47].

#### Depressive symptoms

Depressive symptoms were measured using the Center for Epidemiological Studies Depression Scale (CES-D). It consists of 10 items with scores ranging from 0 to 60, with higher scores indicating more severe depressive symptoms [48].

#### Health-related quality of life

Health-related quality of life (HRQoL) was measured using the functional assessment of cancer therapy-leukemia *(*FACT*-*Leu)*.* FACT-Leu is a valid measure for patients with acute or chronic leukemia and consists of five subsections: physical well-being, social/family well-being, emotional well-being, and leukemia-specific symptoms. Each question is rated on a five-point Likert scale, and higher scores indicate better HRQoL [49].

#### Cognition

Cognition was measured using the Montreal Cognitive Assessment (MOCA) or MOCA-Blind (if in-person assessment was not possible due to COVID-19 pandemic), with scores ranging from 0–30 to 0–22, respectively [50–52]. Higher scores indicate better cognition.

#### DNA methylation

For DNAm analysis, 1000 ng of DNA was isolated from whole blood and bisulfite converted (converts cytosine to uracil but leaves 5-methylcytosine residues unaffected). DNA methylation microarray assay was performed using the Illumina Infinium^®^ Methylation EPIC Array platform, an oligonucleotide array that interrogates > 850,000 CpG dinucleotides per sample. Assays were performed by Roswell Park Genomics Shared Resource laboratory per manufacturer’s protocol. The raw data were processed by the R package “minfi” [53] and converted to methylation ß-values ranging from 0 (unmethylated) to 1 (fully methylated) to represent the methylation level of each CpG site. Potential residue batch effects were inferred from the data using a Surrogate Variable Analysis [54], and the ComBat algorithm was used for correction [54]. The final data were supplied to the online DNAm age calculators (https://dnamage.genetics.ucla.edu/). GrimAge [20], PhenoAge [23], Horvath Age [21, 22], Hannum Age [24], and DunedinPACE. We focused on DNAm GrimAge, DNAm PhenoAge, and DunedinPACE [21].

### Analyses

We used descriptive statistics to summarize our study sample, clinical measures, and accelerated DNAm age [Horvath Age, Hannum Age, GrimAge, PhenoAge, and DunedinPACE, as well as intrinsic (IEAA) and extrinsic (EEAA) epigenetic age acceleration; EEAA adjusts for blood cell proportions whereas IEAA is independent of blood cell proportions). Accelerated Horvath Age, Hannum Age, GrimAge, and PhenoAge were calculated from the difference between DNAm age and chronologic age, with positive values suggesting faster aging and negative values reflecting slower aging. DunedinPACE was calculated using “DunedinPACE” R package [32]. To assess whether changes in DNAm ages from baseline to post-intervention were significantly different from zero, we used paired t-tests or Wilcoxon signed rank tests when differences were not normally distributed. For relationships between accelerated ages and measures, we focused specifically on the second generation epigenetic clocks (GrimAge and PhenoAge) and DunedinPACE. To assess the relationships between accelerated DNAm ages and measures, we used Spearman’s rank correlation coefficient.

Given our small sample size and the exploratory nature of our study, we pre-specified *α* = 0.10 (2-tailed) for hypothesis testing to indicate a significance threshold of interest for future studies. For the same reasons, we did not do multiple testing. We used the R to calculate DunedinPACE and SAS v.9.4 (SAS Institute Inc., Cary, NC) to perform the remaining analyses.

## Results

### Demographics

We previously published the demographics and clinical characteristics of the 25 participants [41]. Twenty patients had complete DNAm data at baseline and post-intervention and were included in the analysis (Table 1). Mean age of the 20 participants was 71.2 (SD 4.8, range 62–80), 65% were males, 90% were white, 75% had Karnofsky Performance Status 70–100, and 55% had acute myeloid leukemia. Table 2 shows the disease status and blood counts at baseline and post-intervention.

**Table 1:** Demographics and clinical characteristics

Variables		N = 20
Age in years, mean (SD, range)		71.2 (4.8, 62–80)
Gender, *n* (%)	Male	13 (65.0)
	Female	7 (35.0)
Race, *n* (%)	White	18 (90.0)
	Black or African American	1 (5.0)
	Prefer not to say	1 (5.0)
Ethnicity, *n* (%)	Not Hispanic or Latino	19 (95.0)
	Prefer not to say	1 (5.0)
Marital status, *n* (%)	Married	13 (65.0)
	Divorced or widowed	2 (10.0)
	Single	5 (25.0)
Education, *n* (%)	High school or below	2 (10.0)
	At least some college	6 (30.0)
	College graduate	5 (25.0)
	Postgraduate level	6 (30.0)
	Prefer not to say	1 (5.0)
Karnofsky performance status, *n* (%)	90–100	3 (15.0)
	70–80	12 (60.0)
	50–60	5 (25.0)
Diagnosis, *n* (%)	AML	11 (55.0)
	MDS	8 (40.0)
	MDS/myeloproliferative neoplasm overlap syndromes	1 (5.0)
Treatment, *n* (%)	HMA combination treatment (e.g., venetoclax)	11 (55.0)
	HMA only	7 (35.0)
	Other*	2 (10.0)
Chemotherapy cycle at initiation of intervention, *n* (%)*	1	3 (15.0)
	2	9 (45.0)
	3	4 (20.0)
	≥ 4	4 (20.0)

AML, acute myeloid leukemia; HMA, hypomethylating agent; MDS, myelodysplastic syndrome

^*^1 received gilteritinib and 1 received low dose cytarabine and venetoclax

**Table 2:** Disease status and blood counts at baseline and post-intervention

Disease status		Baseline	Post-intervention	Change	*P*
Disease status, *n* (%)					0.81^
Active MDS		7 (35.0)	7 (35.0)		
Active AML		4 (20.0)	4 (20.0)		
Remission		7 (35.0)	8 (40.0)		
Unable to be determined		2 (10.0)	1 (5.0)		
Blood counts					
White blood cell, thousand/uL	Mean (SD)	2.91 (2.70)	3.05 (2.23)	0.14 (2.41)	
	Median (IQR)	1.95 (3.80)	2.25 (4.30)	0.35 (1.95)	0.45*
Absolute neutrophil count, thousand/uL	Mean (SD)	1.59 (1.98)	1.45 (1.63)	− 0.14 (1.50)	
	Median (IQR)	0.65 (2.55)	0.85 (2.65)	− 0.00 (1.20)	0.91*
Absolute monocyte count	Mean (SD)	0.28 (0.35)	0.21 (0.28)	− 0.07 (0.28)	
	Median (IQR)	0.10 (0.50)	0.10 (0.35)	− 0.02 (1.30)	0.29*
Absolute lymphocyte count	Mean (SD)	0.90 (0.44)	1.00 (0.44)	0.09 (0.39)	
	Median (IQR)	0.95 (0.75)	1.00 (0.65)	0.10 (0.40)	0.17*
Hemoglobin, g/dL	Mean (SD)	9.31 (2.32)	9.05 (2.97)	− 0.26 (1.61)	
	Median (IQR)	8.45 (2.15)	8.95 (3.05)	− 0.30 (1.20)	0.30*
Platelets, thousand/uL	Mean (SD)	148.98 (110.82)	116.97 (93.63)	− 32.02 (112.05)	
	Median (IQR)	143.50 (121.00)	92.50 (130.00)	− 13.00 (66.50)	0.22*^

AML, acute myeloid leukemia; HMA, hypomethylating agent; MDS, myelodysplastic syndrome

^*P* value from Bowker exact symmetry test

^*^P value from Wilcoxon signed rank test

At baseline, patients walked on average 3289.4 (SD 2056.0, *n* = 18) steps per day. At post-intervention, patients walked 3649.1 (SD 2651.8, *n* = 18) daily steps. Patients reported performing resistance band exercises for a mean duration of 26.4 (SD 10.21, *n* = 19) minutes/day, 3.0 (SD 2.3, *n* = 19) days/week, and they rated their perceived exertion at 3.4 (SD 1.2, *n* = 18) on a 1–10 Likert scale, indicating low intensity.

### DNAm ages at baseline and post-intervention

DNAm ages are shown in Table 3 and Additional file 1: Fig. S1. At baseline, mean GrimAge was 73.2 years [SD 6.8; accelerated GrimAge = 1.5 years (SD 5.4)] and mean PhenoAge was 58.2 years [SD 9.7; accelerated PhenoAge = − 13.4 years (SD 9.0)]. Mean DunedinPACE was 1.2 years (SD 0.3). GrimAge and PhenoAge were stable from baseline to post-intervention [median change for GrimAge = − 1.4 years (interquartile range (IQR) 4.5), *p* = 0.17 and median change for Pheno Age = − 1.4 years (IQR 12.4), *p* = 0.35] (Table 2). Additional file 2: Fig. S2 shows the individual-level changes and by treatment types (HMA combination treatment, HMA only, and others). No consistent pattern of changes in DNA methylation ages are noted with treatment types. GrimAge decreased in 14 of 20 patients, and PhenoAge decreased in 13 of 20. Median DunedinPACE remained stable [median change = − 0.1 (IQR 0.2), *p* = 0.47]; DunedinPACE decreased in 14 of 20 patients.

**Table 3:** DNA methylation ages at baseline and post-intervention

Baseline ages (*N* = 20)	Statistics	Baseline	Post-intervention	Change	P*
Chronological age	Mean (SD)	71.7 (4.9)	71.9 (4.8)		
	Median (IQR)	72.4 (6.5)	72.6 (6.5)		
Horvath age	Mean (SD)	70.8 (9.3)	70.3 (9.7)	− 0.5 (5.6)	
	Median (IQR)	69.6 (13.4)	68.7 (15.1)	− 0.8 (7.4)	0.73
Hannum age	Mean (SD)	58.4 (9.1)	58.7 (8.6)	0.3 (10.2)	
	Median (IQR)	59.1 (7.5)	57.6 (11.4)	− 0.7 (10.8)	0.57
IEAA	Mean (SD)	− 1.0 (7.9)	− 1.6 (7.8)	− 0.7 (4.1)	
	Median (IQR)	− 3.0 (10.3)	− 1.8 (11.4)	− 1.0 (5.3)	0.43
EEAA	Mean (SD)	− 1.2 (10.8)	− 1.0 (11.2)	0.2 (12.5)	
	Median (IQR)	− 0.6 (14.1)	− 0.1 (15.8)	− 0.7 (13.7)	0.81
GrimAge	Mean (SD)	73.2 (6.8)	72.5 (5.7)	− 0.7 (4.7)	
	Median (IQR)	73.5 (8.1)	72.3 (6.9)	− 1.4 (4.5)	0.17
PhenoAge	Mean (SD)	58.2 (9.7)	57.4 (8.5)	− 0.8 (12.1)	
	Median (IQR)	57.2 (14.9)	57.0 (6.9)	− 1.4 (12.4)	0.35
DunedinPACE	Mean (SD)	1.2 (0.3)	1.2 (0.2)	0.02 (0.34)	
	Median (IQR)	1.1 (0.4)	1.2 (0.3)	− 0.1 (0.2)	0.47

EEAA, extrinsic epigenetic age acceleration; IEAA, intrinsic epigenetic age acceleration

^*^P value from Wilcoxon signed rank test

### Associations of accelerated DNAm ages with physical, psychological, and cognitive functions

Table 4 shows the outcomes at baseline and post-intervention among those who completed these measures at both time points (*n* = 20). The SPPB, BFI, CES-D, and FACT-Leu data have been previously reported [41]. Overall, physical, psychological, and cognitive functions were stable from baseline to post-intervention. When clinically meaningful cut-off scores were utilized, lower percentages of participants had physical [except for ADL and IADL (IADL), psychological, and cognitive impairments at post-intervention than at baseline.

**Table 4:** Outcomes at baseline and post-intervention for patients with complete DNA methylation data

*N* = 20	Statistic	Baseline	Post-intervention	Change from baseline to post-intervention	P^e^
Short Physical Performance Battery (SPPB)^a^	Mean (SD)	9.00 (1.78)	9.25 (2.53)	0.25 (1.62)	0.66
	Median (IQR)	9.00 (2.50)	10.00 (3.00)	1.00 (2.00)	
SPPB < 10 is considered impaired	N (%)	11 (55.0%)	9 (45.0%)		0.69
Virtual SPPB^a, c^	Mean (SD)	8.07 (2.94)	8.38 (2.93)	0.08 (2.35)	0.73
	Median (IQR)	9.00 (5.00)	9.00 (6.00)	0.50 (1.50)	
Virtual SPPB < 10 is considered impaired	N (%)	9 (75.0%)	8 (66.7%)		1
6-minute walk test, in meters^a^	Mean (SD)	360.20 (135.93)	334.49 (187.98)	− 25.71 (151.50)	0.46
	Median (IQR)	377.04 (172.21)	392.89 (268.99)	7.16 (114.76)	
Grip strength, in kilograms^a^	Mean (SD)	25.37 (9.74)	25.86 (9.04)	0.49 (3.18)	0.50
	Median (IQR)	25.50 (13.63)	25.46 (13.88)	0.58 (4.52)	
< 25.8 is considered impaired in men and < 17.4 is considered impaired in women	N (%)	10 (50.0%)	8 (40.0%)		0.63
Activities of Daily Living (ADL)^a^	Mean (SD)	5.85 (0.49)	5.85 (0.49)	0.00 (0.32)	1
	Median (IQR)	6.00 (0.00)	6.00 (0.00)	0.00 (0.00)	
< 6 is considered impaired	N (%)	2 (10.0%)	2 (10.0%)		1
Instrumental IADL^a^	Mean (SD)	12.50 (1.88)	12.30 (2.00)	− 0.20 (1.15)	0.56
	Median (IQR)	13.00 (2.00)	13.00 (4.00)	0.00 (0.00)	
< 14 is considered impaired	N (%)	2 (10.0%)	2 (10.0%)		1
Brief Fatigue Inventory^b^	Mean (SD)	28.45 (20.25)	23.85 (19.60)	− 4.60 (16.86)	0.24
	Median (IQR)	25.00 (27.00)	15.50 (39.00)	− 2.00 (23.50)	
Center for Epidemiologic Studies Depression^b^	Mean (SD)	12.10 (7.71)	11.50 (8.14)	− 0.60 (6.34)	0.68
	Median (IQR)	11.50 (13.50)	10.50 (9.50)	0.00 (6.50)	
> 15 is considered impaired	N (%)	6 (30.0%)	4 (20.0%)		0.63
Functional Assessment of Cancer Therapy-Leukemia^a^	Mean (SD)	125.20 (21.67)	127.20 (24.02)	2.00 (13.28)	0.51
	Median (IQR)	126.92 (25.83)	133.50 (28.17)	1.83 (20.25)	
Montreal Cognitive Assessment (MOCA)^a,d^	Mean (SD)	25.29 (3.48)	25.76 (3.05)	0.47 (3.00)	0.53
	Median (IQR)	26.00 (5.00)	27.00 (3.00)	0.00 (3.00)	
< 26 is considered impaired	N (%)	7 (41.2%)	6 (35.3%)		1.00

^a^Higher is better

^b^Lower is better

^c^12 patients

^d^17 patients

^e^P value from McNemar’s test for categorical (impairment) and paired t test or Wilcoxon signed rank test for continuous variables

**Fig. 1:**
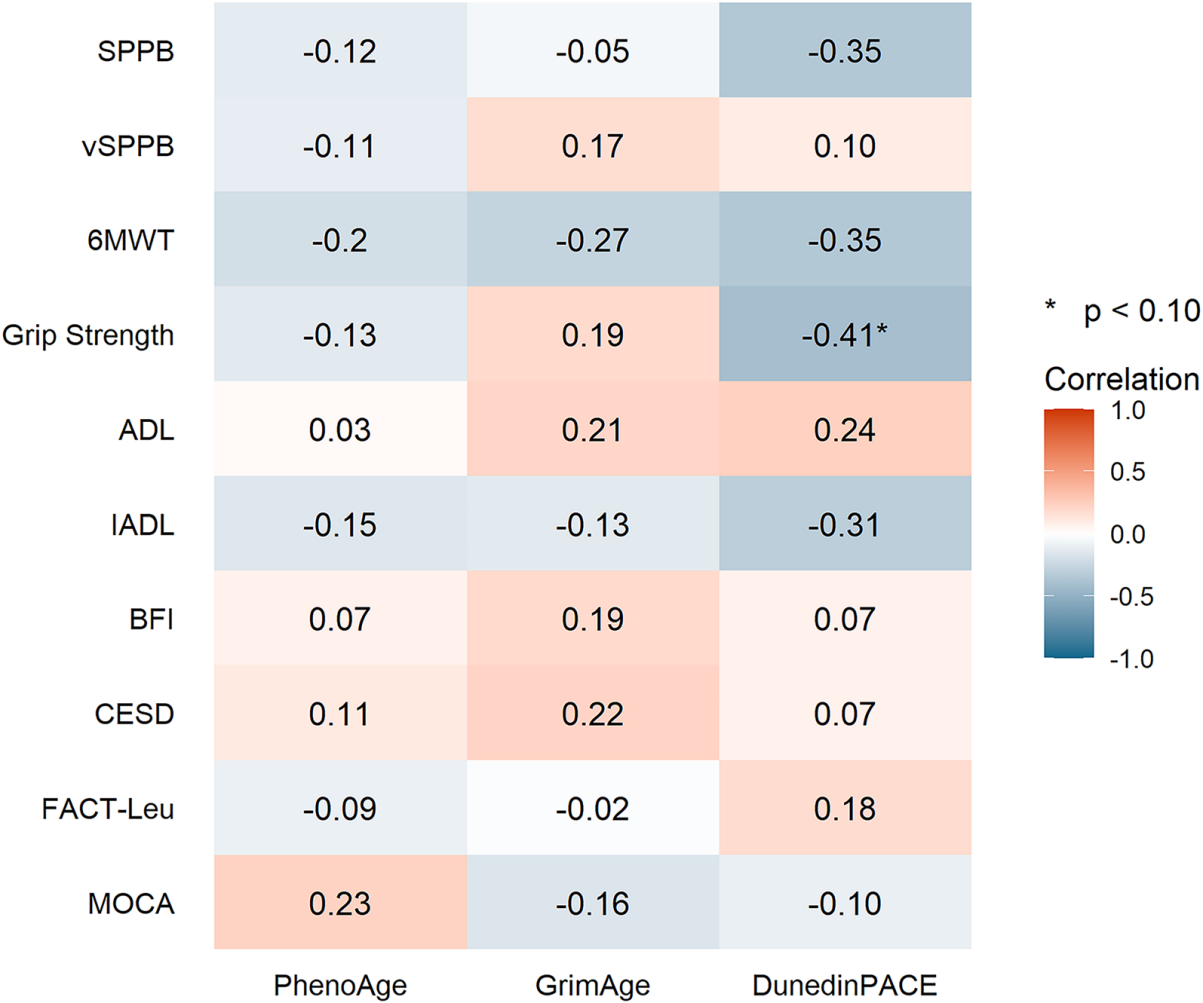
Correlation between baseline accelerated DNA methylation age and baseline physical, psychological, and cognitive functions. 6MWT, 6-minute walk test; ADL, Activities of Daily Living; BFI, Brief Fatigue Inventory; CES-D, Center for Epidemiological Studies Depression; FACT-Leu, Functional Assessment of Cancer Therapy-Leukemia; IADL, Instrumental Activities of Daily Living; SPPB—Short Physical Performance Battery; vSPPB—Virtual Short Physical Performance Battery

At baseline, DunedinPACE was inversely correlated with grip strength (r = − 0.41, *p* = 0.08) (Fig. 1).

### Associations of change in DNAm age with changes in physical, psychological, and cognitive functions from baseline to post-intervention

**Fig. 2:**
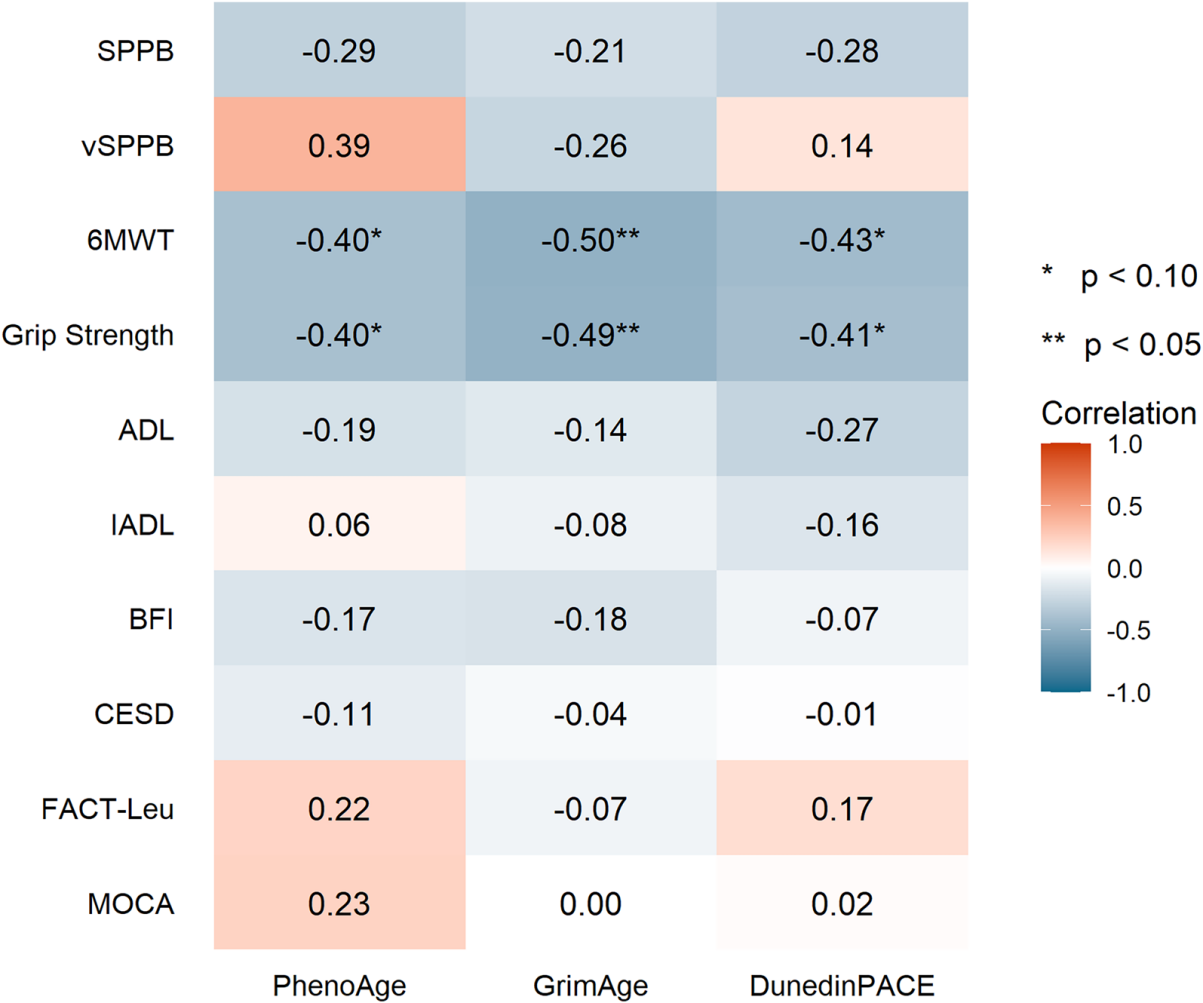
Correlation between changes in accelerated DNA methylation age and changes in physical, psychological, and cognitive functions. Δ; changes from baseline to post-intervention; 6MWT, 6-minute walk test; ADL, Activities of Daily Living; BFI, Brief Fatigue Inventory; CES-D, Center for Epidemiological Studies Depression; FACT-Leu, Functional Assessment of Cancer Therapy-Leukemia; IADL, Instrumental Activities of Daily Living; SPPB—Short Physical Performance Battery; vSPPB—Virtual Short Physical Performance Battery

From baseline to post-intervention, the change in accelerated DNAm ages, as determined using GrimAge, PhenoAge, and DunedinPace were correlated with the change in distance walked on 6-minute walk test (6MWT) and grip strength. Decreases in accelerated GrimAge (r = − 0.47, *p* = 0.04), accelerated PhenoAge (r = − 0.38, *p* = 0.09), and DunedinPace (r = − 0.43, *p* = 0.06) were correlated with increases in distance walked on 6MWT (Fig. 2). Similarly, decreases in accelerated GrimAge (r = − 0.49, *p* = 0.03), PhenoAge (r = − 0.42, *p* = 0.07), and DunedinPace (r = − 0.41, *p* = 0.07) were correlated with increases in in grip strength.

### DNAm ages and exercise levels

To explore changes in exercise levels and changes in DNAm age, we stratified the group by the degree to which their steps and resistance minutes increased from baseline to post-intervention (> median vs ≤ median; Table 5). Compared to those who had an increase in steps ≤ median from baseline to post-intervention, patients who had an increase in steps > median showed a greater decrease in DNAm age. For example, among those who had increased steps > median, change in median GrimAge from baseline to post-intervention was -2.66 (IQR 4.06). Among those who had an increased in steps ≤ median, change in median GrimAge from baseline to post-intervention was + 0.79 (IQR 2.85). However, no consistent association between changes in DNA age and minutes of resistance exercise was observed (Table 4). Additional file 3: Fig. S3 shows the changes in DNAm age by steps at an individual level.

**Table 5:** Subgroup analysis evaluating change in accelerated DNA methylation age from baseline to post-intervention compared by change in daily steps or minutes of resistance exercise

ΔDNAm accelerated age	Statistic	Increase in steps from baseline to post-intervention (≥ median) n = 9	Increase in steps from baseline to post-intervention (< median) n = 9	P*	Increase in minutes from baseline to post-intervention (≥ median) n = 9	Increase in minutes from baseline to post-intervention (< median) n = 10	P*
ΔPhenoAge	Mean (SD)	− 2.83 (8.89)	− 0.82 (10.56)		2.86 (15.02)	− 3.15 (9.27)	
	Median (IQR)	− 5.58 (11.63)	− 0.05 (7.90)	0.79	− 1.55 (12.56)	− 3.08 (17.52)	0.71
ΔGrimAge	Mean (SD)	− 1.98 (3.22)	1.83 (5.68)		− 0.10 (5.42)	− 0.04 (4.37)	
	Median (IQR)	− 2.66 (4.06)	0.79 (2.85)	0.08	− 0.32 (2.65)	− 0.30 (4.93)	0.90
ΔDunedinPACE	Mean (SD)	− 0.13 (0.28)	0.08 (0.29)		0.10 (0.40)	− 0.05 (0.31)	
	Median (IQR)	− 0.18 (0.27)	− 0.03 (0.13)	0.13	− 0.05 (0.15)	− 0.07 (0.22)	0.49

^*^P value from Wilcoxon two-sample test comparing median change in accelerated DNA age for those who increased by ≥ median steps or resistance minutes to those who increased by < median steps or resistance minutes

## Discussion

In this single-arm pilot study, we demonstrated that it was feasible to evaluate DNAm ages using blood samples collected as part of a mHealth exercise trial among older adults with myeloid malignancies. We evaluated the relationship between accelerated DNAm age and physical, psychological, and cognitive functions in older adults with myeloid malignancies. Examination of correlations between accelerated DNAm ages and clinical measures at baseline revealed that DunedinPACE was inversely correlated with grip strength. We showed that after a mhealth exercise intervention (over two cycles of treatment or approximately 8–12 weeks), DNAm age measured via GrimAge and PhenoAge were stable from baseline to post-intervention. Nevertheless, from baseline to post-intervention, decreases in accelerated GrimAge, accelerated PhenoAge, DunedinPACE, were correlated with increase in both distance walked on 6MWT and in grip strength.

Older adults with myeloid malignancies are vulnerable to treatment-related toxicities which can lead to declines in physical, psychological, and cognitive functions, thereby increasing morbidity and mortality. Identifying those at risk using a biomarker such as accelerated DNAm age allows healthcare professionals to warn of declines in functions. Accelerated DNAm age is associated with functional decline in the general population.^20–21^ For example, in a previous cross-sectional study, older adults (aged > 60 years) with accelerated aging (PhenoAge) were found to have decreased physical performance (measured using the 6MWT) [55]. In a longitudinal study of middle-aged urban adults, accelerated DNAm age was associated with diminished performance on visual memory/visuoconstructive ability tests and attention/processing speed [28]. In a cross-sectional analysis of older adults (> 70 years old), accelerated DNAm age (Horvath Age) was associated with poorer lung function, cognitive function, and grip strength [56]. In the cancer population, a longitudinal study of patients with head and neck cancer undergoing radiation therapy demonstrated that those who experienced severe fatigue had higher accelerated DNAm age (PhenoAge) by 3.1 years compared to those who did not [57]. Our study supports these studies by demonstrating that DunedinPACE is inversely correlated with grip strength among older adults with myeloid malignancies.

Behavioral interventions, such as the mHealth exercise intervention evaluated here, may ameliorate treatment-related toxicities and slow the rate of accelerated aging. After an 8-week exercise intervention in older patients with myeloid malignancies, we found that DNAm age was generally unchanged. While we do not have a control arm for comparison, a previous study demonstrated among patients with breast cancer, radiation and chemotherapy lead to accelerated DNAm age from pre- to post-treatment by 6.2–25.6 years [30]. In a mouse study, DNAm age measured from skeletal muscle was younger in mice who were subjected to endurance exercise training compared to their sedentary counterparts [58]. Prior population-based studies have also evaluated the relationship between DNAm age and physical activity [39, 60]. For example, Sillanpaa and colleagues explored the association of various levels of physical activity with DNAm age in a cross-sectional study of adults aged 23–69 years. They demonstrated that compared to adults with low activity levels, measured using accelerometers, those with medium activity levels had lower accelerated GrimAge (− 3.20; *p* = 0.04).[60] In a randomized controlled trial, 43 healthy adult men aged 50–72 were assigned to an 8-week behavioral intervention (diet, sleep, exercise, and relaxation guidance, and supplemental probiotics and phytonutrients) versus controls. Participants in the intervention arm had decreased DNAm age (Horvath Age) compared to controls (3.23 years, *p* = 0.018).[61] Finally, in a single-arm pilot study of older adults with hematologic malignancies, Rosko and colleagues demonstrated that PhenoAge decreased in 3 of 10 patients after a 6-month exercise intervention [40].

We demonstrated decreases in accelerated aging were correlated with increases in both distance walked on 6MWT and grip strength. However, the mechanisms by which epigenetic clocks are changed in response to behavioral interventions such as exercise are unclear. DNAm ages are generated from a set of CpG sites, and the methylation levels are a reflection of biological age. These CpG sites reside across the genome, but depending on the platform used to measure methylation, are biased towards promoter regions and may therefore influence expression of certain genes. Previous studies have demonstrated that exercise can lead to hypomethylation and hypermethylation of specific CpG sites, as well as global hypomethylation and hypermethylation [62–67]. Of note, Brown and colleagues showed that exercise-induced DNA methylation modification was stronger among older versus younger individuals, which suggests that exercise may be more effective in slowing accelerated DNAm age in older individuals [68].

Our study has strengths. First, we included older adults with myeloid malignancies, a population not typically studied in clinical trials. Second, we were able to measure DNAm ages prospectively in a clinical trial. Several limitations also should be noted. For example, our sample includes patients with various myeloid malignancies and at different stages and types of treatment (e.g., hypomethylating agents alone or in combination). Therefore, it is difficult to differentiate the effects of the cancer, treatment, and exercise on DNAm ages. Given the small sample size, we were unable to perform subgroup analyses and it may have also limited our ability to detect other associations. Given the evolving treatment landscape for myeloid malignancies and the increasing difficulty in recruiting a homogeneous population, future larger multicenter trials are needed to recruit this population in order to understand the influence of aging, cancer, treatment, and exercise on DNAm ages.

In conclusion, DunedinPACE is inversely correlated with grip strength at baseline. We demonstrated that GrimAge and PhenoAge on average are stable after a mHealth exercise intervention in older adults with myeloid malignancies receiving chemotherapy. Decreases in accelerated PhenoAge and GrimAge as well as decreases in DunedinPACE over 8–12 weeks of exercise are correlated with increased physical performance. Our findings will inform an ongoing pilot randomized controlled trial (clinicaltrials.gov identifier: NCT04981821) testing the effect of the mHealth exercise intervention in older adults with myeloid malignancies, in which we will evaluate the change in DNAm age, comparing the intervention and control arms. Our study supports the use of GrimAge, PhenoAge, and DunedinPACE when measuring accelerated aging as part of an exercise clinical trial.

## Supplementary Information

**Additional file 1: Figure S1.** DNA methylation ages at baseline and post-intervention. **A** Chronological and DNAm Ages at baseline and post intervention and **B** DNAm Age accelerations at baseline and post-intervention.

**Additional file 2: Figure S2.** Changes in DNA methylation ages at the individual patient level and by treatment types.

**Additional file 3: Figure S3.** Changes in DNA methylation ages at the individual patient level and by exercise levels. **A** Daily steps and **B** Minutes of resistance exercises.

## Data Availability

The datasets used and/or analyzed during the current study are available from the corresponding author on reasonable request.

## Abbreviations

AML: Acute myeloid leukemia
MDS: Myelodysplastic syndromes
QoL: Quality of life
DNAm: DNA methylation
GO-EXCAP: Geriatric oncology-Exercise for Cancer Patients
ACSM: American College of Sports Medicine
SPPB: Short Physical Performance Battery
6MWT: 6-minute walk test
ADL: Activities of daily living
IADL: Instrumental activities of daily living
BFI: Brief fatigue inventory
CES-D: Center for Epidemiological Studies Depression Scale
HRQoL: Health-related quality of life
FACT-Leu: Functional assessment of cancer therapy-leukemia
MOCA: Montreal Cognitive Assessment
HMA: Hypomethylating agent
SD: Standard deviation
IQR: Interquartile range
