# Supplementary figures and images for: Exercise and epigenetic ages in older adults with myeloid malignancies
Source: Eur J Med Res. 2023 May 30;28:180. doi: 10.1186/s40001-023-01145-z (PMC10227405; doi:10.1186/s40001-023-01145-z)

A)

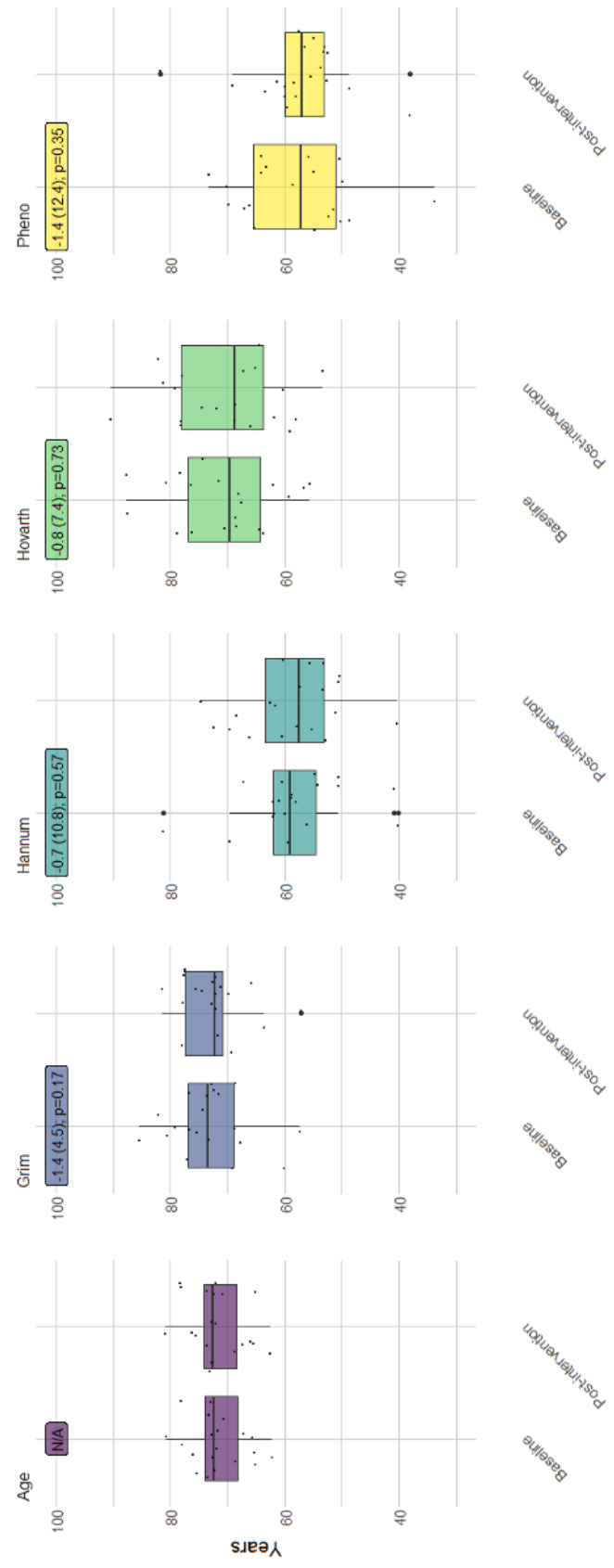

B)

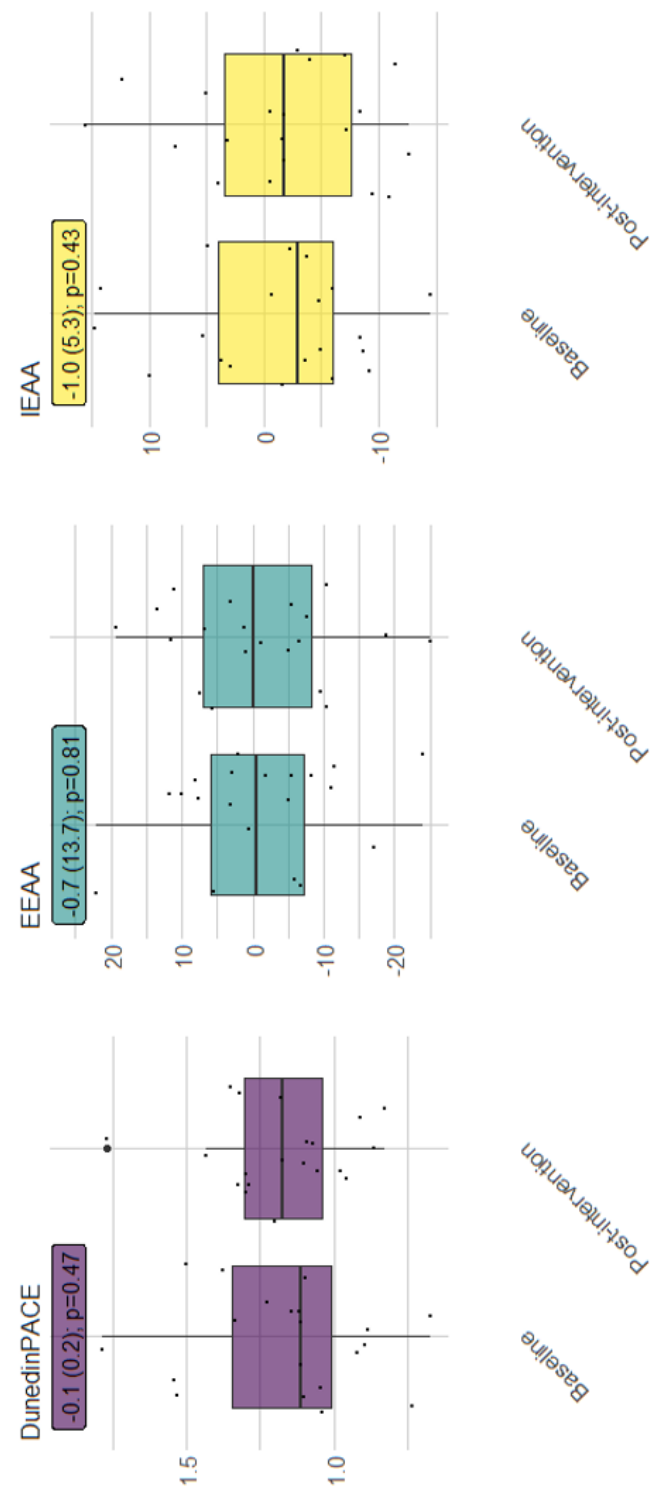

Supplement: Supplementary file 1 — Additional file 1: Figure S1. DNA methylation ages at baseline and post-intervention. A Chronological and DNAm Ages at baseline and post intervention and B DNAm Age accelerations at baseline and post-intervention. [file 40001_2023_1145_MOESM1_ESM.pdf]

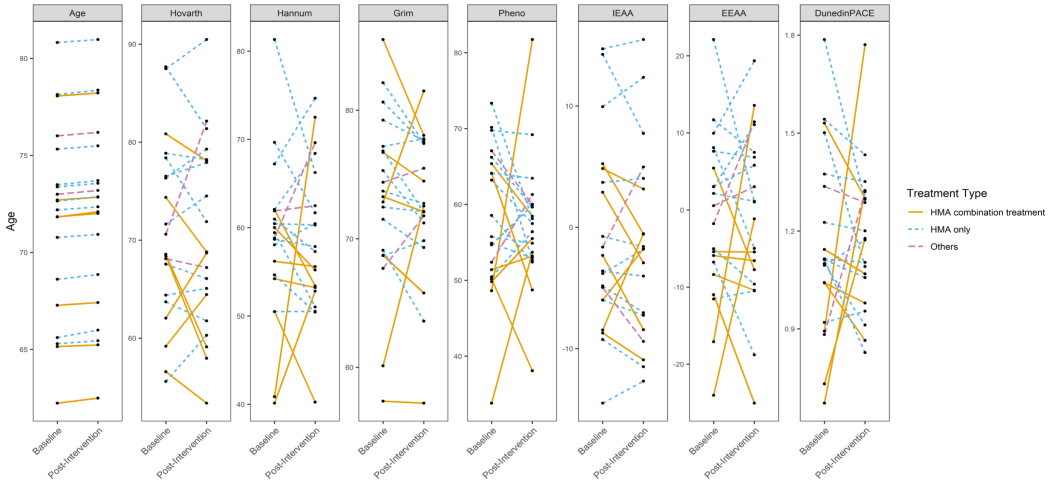

Supplement: Supplementary file 2 — Additional file 2: Figure S2. Changes in DNA methylation ages at the individual patient level and by treatment types. [file 40001_2023_1145_MOESM2_ESM.pdf]

A)

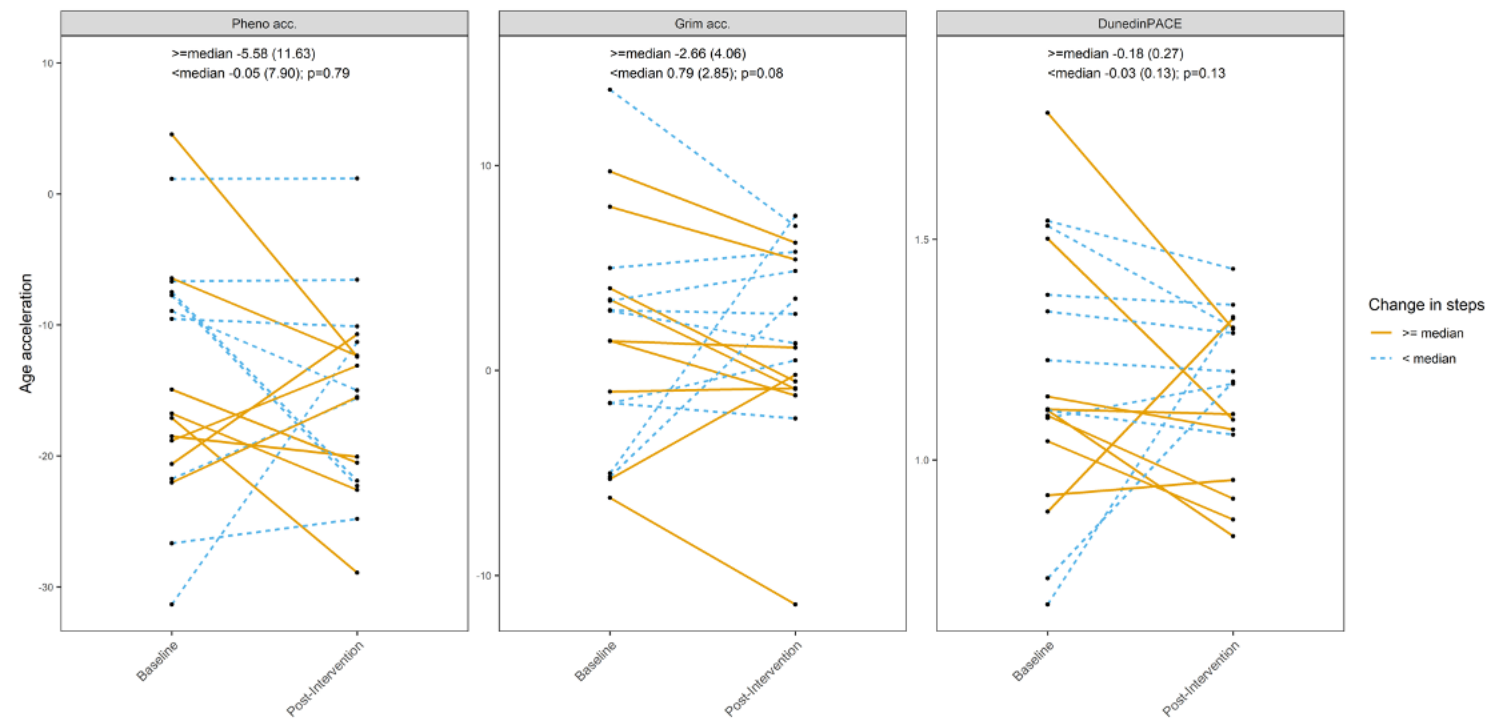

B)

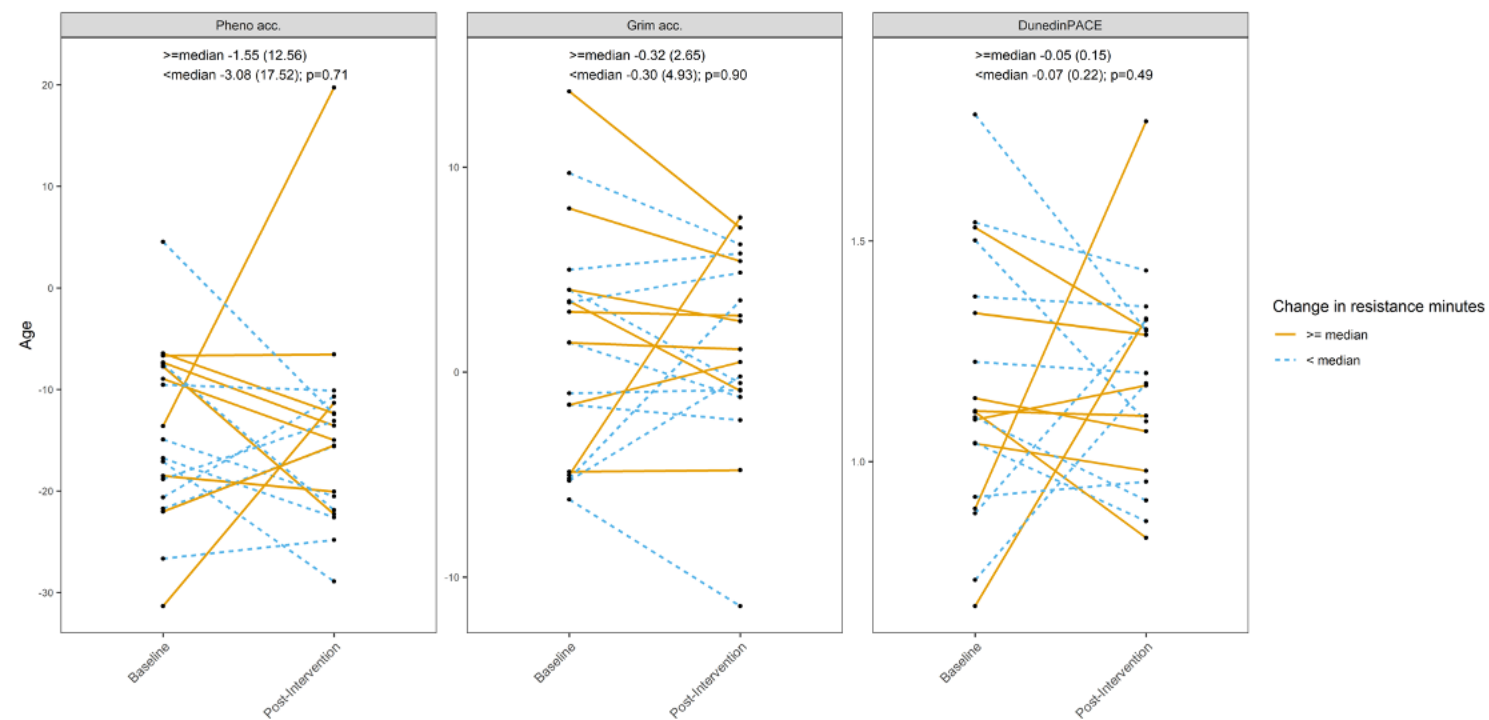

Supplement: Supplementary file 3 — Additional file 3: Figure S3. Changes in DNA methylation ages at the individual patient level and by exercise levels. A Daily steps and B Minutes of resistance exercises. [file 40001_2023_1145_MOESM3_ESM.pdf]
